# The Characters of Second Phases and Texture of Aluminum Alloy Thin-Walled Capsule Welded Joints at Typical HIP Temperature

**DOI:** 10.3390/ma19153202

**Published:** 2026-07-27

**Authors:** Zhanfang Wu, Yazhou Xu, Zhoujin Lv, Xiangyang Li, Dianchun Ju

**Affiliations:** 1 CISRI HIPEX Technology Co., Ltd., Haidian, Beijing 100081, China; wuzhanfang@hipex.cn (Z.W.); lvzhoujin@hipex.cn (Z.L.);; 2 School of Metallurgy Engineering, Jiangsu University of Science and Technology, Zhangjiagang 215600, China; 13235257010@163.com; 3 National Key Laboratory of Metallurgical Intelligent Manufacturing System, China Iron & Steel Research Institute Group Co., Ltd., Beijing 100081, China

**Keywords:** hot isostatic pressing, aluminum alloys, spheroidization, microstructure evolution, crystallographic texture

## Abstract

Aluminum alloy capsules play a critical role in shape control, heat transfer, and pressure transmission during the PM-HIP sintering of Al-based powders. As the weakest part of the capsule, the reliability of the welded joint is crucial for the safe operation of the HIP process and the quality of the final product. This study investigated the effects of two typical HIP temperatures on the evolution of second phases and texture. The results show that: (1) At 400 °C, suppressed Si diffusion retains a continuous, low-melting-point Al-Si eutectic network and needle-like secondary phases in the weld zone, thereby impeding residual stress relief. Compositional segregation in the heat-affected zone weakens grain boundary stability. The texture undergoes only limited recovery, with a strong <100> orientation retained and micro-strain not effectively relieved, restricting joint ductility. (2) At 510 °C, Si is sufficiently spheroidized, forming a bead-like structure. Needle-like second phases transform into globular/short-rod morphologies, disrupting the continuity of the brittle phases. Simultaneously, complete recrystallization is induced, resulting in a randomized texture and significant release of micro-strain, thereby improving microstructural homogeneity and plastic deformation capacity. This study suggests that the internal stress concentration arising from the low-melting-point eutectic phase and strong texture poses a failure risk for the capsule. Therefore, employing the 510 °C HIP process to achieve second-phase spheroidization and texture weakening can significantly mitigate this failure risk.

## 1. Introduction

**Figure 1:**
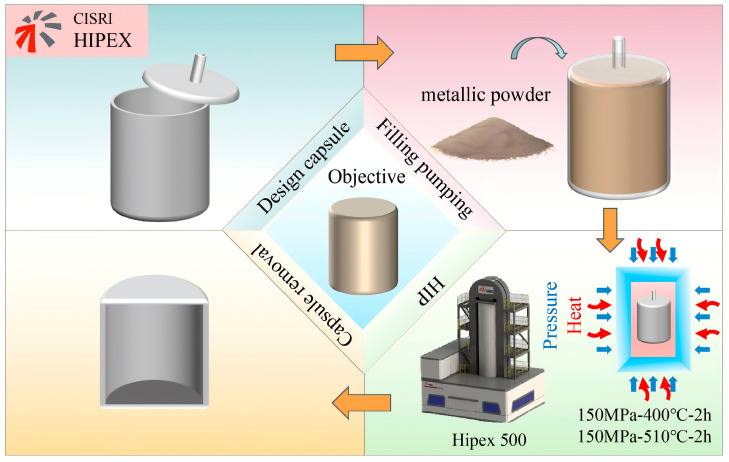
Schematic illustration of the PM-HIP process for the Al capsule, showing the encapsulation of Al-based powder, sealing by welding, and subsequent densification under simultaneous high temperature and isostatic pressure.

Hot Isostatic Pressing (HIP) is an advanced material densification and microstructural modification technology that combines high temperature and high pressure [1,2,3,4,5]. The Powder Metallurgy Hot Isostatic Pressing (PM-HIP) process using capsules, compared to the conventional post-processing of formed components via HIP, enables near-net-shape forming while achieving a texture-free and uniform microstructure [6,7,8]. Current research has focused on stainless steel capsules [9,10,11] and other powder materials for HIP near-net shaping [6,9,12,13], However, studies on aluminum alloy capsules are still lacking. For Al-based powders, thin-walled capsules made of a compositionally similar aluminum alloy are typically used for encapsulation. Such capsules offer a good matching coefficient of thermal expansion with the internal metal powder, effectively reducing thermal stress, and are also convenient for subsequent removal by machining [14]. 1060Al, owing to its excellent ductility, outstanding thermal conductivity, and low cost, is a common choice for such capsules. The schematic of the PM-HIP process is shown in Figure 1.

Capsules are typically manufactured via welding; therefore, the quality of the welded joint determines the shape control effect during HIP and the final product quality [12,13,15]. Aluminum alloy welded joints commonly face problems of microstructural degradation and performance deterioration at high temperatures: thermal cycles can easily lead to coarsening and softening of the weld zone (WZ) and heat-affected zone (HAZ) microstructure, causing a significant drop in joint strength [16,17,18]; the inherent Al-Si low-melting-point eutectic (melting point ~577 °C) in the weld may locally melt at high temperatures, weakening grain boundary cohesion and inducing solidification cracks [19,20]; the strong <100> preferred orientation texture formed by directional solidification in the molten pool can exacerbate the anisotropy of the joint’s mechanical properties, while needle-like second phases act as stress concentration sites, promoting crack initiation and propagation [21]. These issues become more complex under the thermo-mechanical coupling of HIP: although isostatic pressure can partially close pores [22], it can easily induce redistribution of residual liquid phase, coarsening of detrimental phase precipitation, grain boundary migration, recrystallization, and texture reconstruction, altering the original failure mode of the joint [23,24].

Traditional weld quality optimization focuses on adjusting welding parameters to control initial defects. However, for post-weld joints, the HIP process parameters themselves exhibit significant potential for microstructural modification [1,25,26]. Notably, the HIP temperature has a distinct dual effect: appropriately increasing the temperature can promote the spheroidization of the low-melting-point Al-Si eutectic network into a bead-like structure, transform needle-like second phases into short-rod/globular morphologies, simultaneously eliminate the strong texture, release micro-strain, and reduce stress concentration. However, excessively high temperatures may cause grain growth and joint softening [26,27,28]. Nevertheless, systematic research on the microstructural evolution, second-phase segregation, and texture evolution of 1060Al capsule TIG welded joints under different HIP temperatures is still lacking.

This study, guided by the core objective of enhancing reliability and cost-effectiveness of thin-walled capsules in industrial applications, focuses on the influence of HIP temperature on the microstructure evolution and second-phase segregation behavior of 1060Al capsule TIG welded joints. By comparing the microstructural characteristics, elemental distribution, second-phase morphology, and mechanical properties of the joints under different HIP temperatures, the failure risk of the welded joints and their impact on the overall capsule reliability are comprehensively evaluated. Based on these findings, the feasibility of a capsule wall thickness reduction design is discussed. This research aims to provide theoretical support for the thin-wall design, cost control, and production efficiency improvement of 1060Al capsules in industrial manufacturing, as well as to offer a scientific basis for optimizing HIP process parameters.

## 2. Experimental Methods

### 2.1. Specimen Preparation

**Figure 2:**
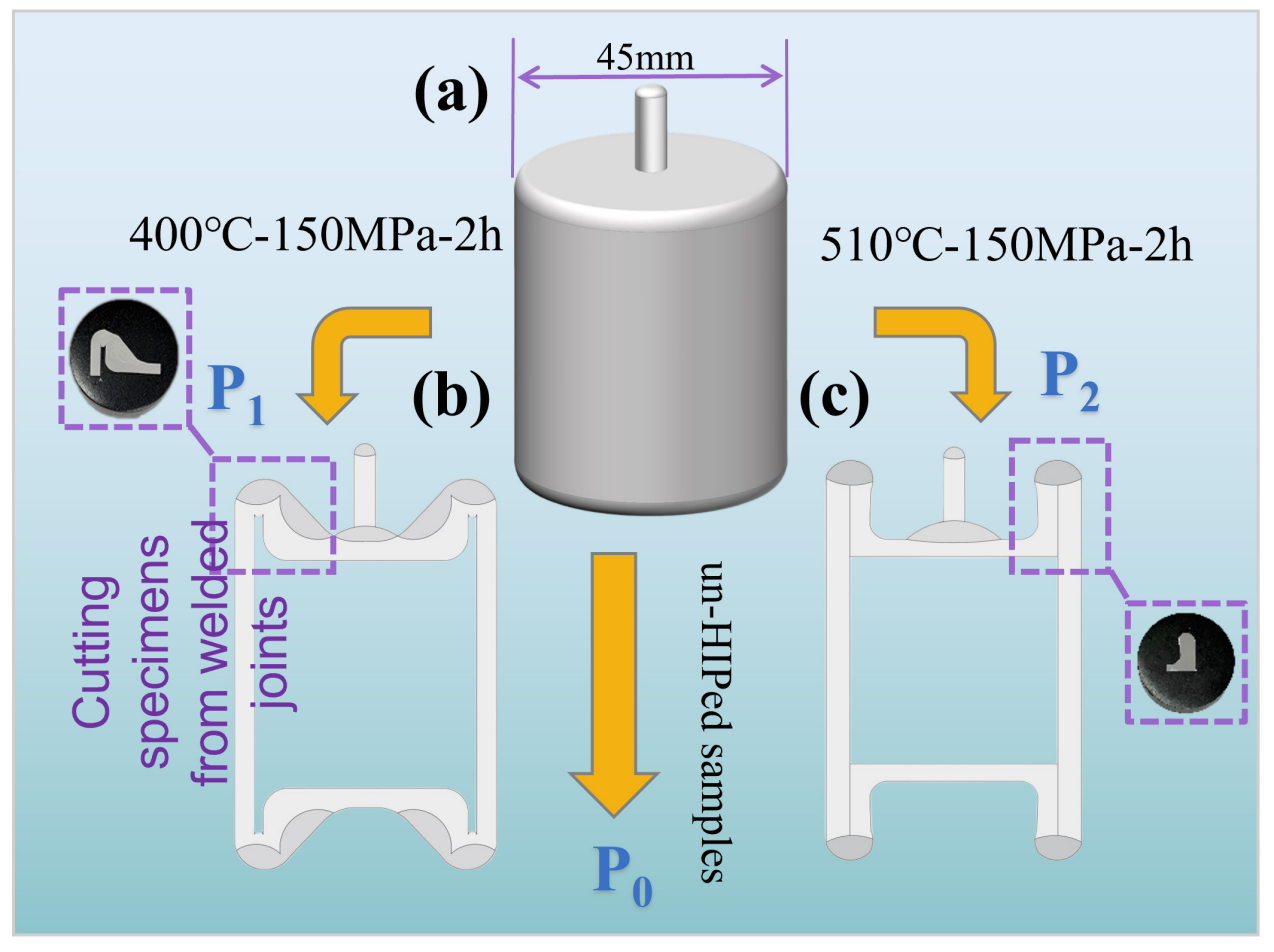
Schematics of (**a**) the as-welded container without HIP (diameter 45 mm, wall thickness 2 mm); (**b**) the welded joint under the P_1_ condition (400 °C-150 MPa-2 h); (**c**) the welded joint under the P_2_ condition (510 °C-150 MPa-2 h).

Thin-walled 1060Al capsules with a wall thickness of 2 mm were prepared using manual Gas Tungsten Arc Welding (GTAW) with a welding current of 110–130 A and a welding voltage of 12–18 V. The chemical compositions of the 1060Al base metal (Baojing Aluminum Industry, Yangzhou, China) and ER1070 filler wire (Sanzhong Weld, Changzhou, China) used are shown in Table 1. The welded capsule without HIP treatment was designated as sample P_0_ (Figure 2a). The capsules were subjected to different HIP process parameters to obtain two types of welded joint samples, whose macroscopic morphologies are shown in Figure 2b and Figure 2c, respectively. The detailed HIP process parameters for samples P_1_ and P_2_ are listed in Table 2.

**Figure 3:**
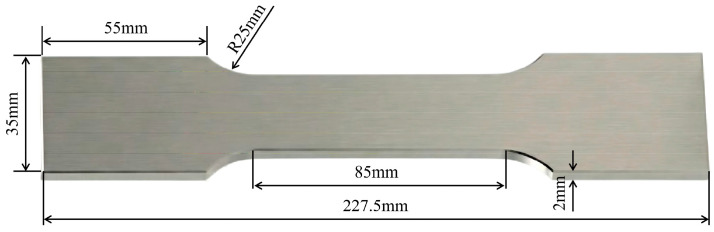
Schematic illustration of the high-temperature tensile specimen dimensions, fabricated with welding parameters identical to those used for capsule preparation to verify the mechanical behavior of the capsule during HIP service.

To verify the mechanical behavior of the capsule during high-temperature HIP service, high-temperature tensile specimens were fabricated using welding parameters identical to those for the capsule preparation. The specific dimensions are shown in Figure 3. The specimens were designated P_W1_ and P_W2_, and their high-temperature tensile test temperatures corresponded to the HIP treatment temperatures of samples P_1_ and P_2_, respectively. The detailed HIP process parameters for samples P_1_ and P_2_ are listed in Table 2, where 400 °C and 510 °C were selected because they produce contrasting regimes of second-phase evolution and texture response in the welded joint, enabling a systematic comparative evaluation of joint failure risk.

The HIP treatment was performed using a CISRI-HIPEX 1850 (HIPEX, Beijing, China), which offers a maximum pressure of 200 MPa and a maximum temperature of 2000 °C. The temperature inside the HIP furnace was monitored using Φ0.20 mm Type C thermocouple wires (WRe5/26) positioned at the bottom of the loaded charge, with a measurement accuracy of ±5 °C, ensuring uniform temperature distribution during the 2 h isothermal holding stage.

Metallographic cross-sectional specimens, 2–3 mm thick, were sectioned from the welded joint region. The specimens were sequentially ground using 1000#, 1500#, and 2000# sandpaper, followed by mechanical polishing: initially rough polished with W2.5 and W1.5 water-soluble diamond paste, and finally fine polished to a mirror finish with a 0.05 μm diamond suspension. After polishing, the base metal and weld regions were etched using Keller’s reagent (HF:HCl:HNO_3_:H_2_O = 1:1.5:2.5:95, by volume) to reveal a clear microstructure.

### 2.2. Material Characterization and Testing

An optical microscope (ZEISS Axio Scope.A1, ZEISS, Oberkochen, Germany) was used to observe microscopic defects and microstructure before and after etching, recording morphology and defect features. A Zwick Z100HT high-temperature tensile testing machine (Zwick, Ulm, Germany) was used to perform high-temperature tensile tests on P_W1_ and P_W2_ specimens at a strain rate of 3.0 × 10^−4^ s^−1^ to evaluate the high-temperature mechanical properties of the welded joints. Microhardness testing was conducted using an HVS-1000A microhardness tester (Laizhou Huayin Testing Instrument, Laizhou, China) with an HV_0.05_ load and 0.1 mm spacing.

An EM30 scanning electron microscope (SEM, COXEM, Daejeon, Republic of Korea) was used to characterize the elemental composition of the welded joints, analyzing Si element distribution and differences in second phases. An EBSD detector (Oxford Instruments, Abingdon, UK) attached to a JSM-6510LA SEM (JEOL, Tokyo, Japan) was used for EBSD analysis with a step size of 4 μm to analyze grain size, dislocation density, and crystallographic texture of the welded joints, providing a microstructural basis for performance analysis.

## 3. Results and Discussion

### 3.1. Macroscopic Deformation and Pore Healing of Capsules

During the HIP process, significant differences in material deformation exist under different process parameters. As shown in Figure 2b,c, compared to the P_1_ process (400 °C), the welded joint obtained from the P_2_ process (510 °C) exhibited more pronounced macroscopic plastic deformation perpendicular to the capsule wall direction.

Although the deformation trend of the thin sheet in various radial directions was generally similar for both P_1_ and P_2_ processes, the capsule end cap and sidewall made contact under compression during the P_2_ process HIP, resulting in greater deformation of its welded joint compared to the P_1_ process. This phenomenon is attributed to the welded joint being the weakest part of the entire capsule; higher temperatures cause greater softening, making it more prone to plastic flow under a 150 MPa isostatic pressure [27,29]. Nevertheless, the capsule sidewall under the P_2_ process maintained a good geometric shape, indicating sufficient shape control capability over the filled powder.

The macroscopic plastic deformation generated during the HIP process drives the contact of pore interfaces and activates subsequent diffusion-based healing mechanisms [22,23]. The overall macroscopic plastic deformation of the capsule is a prerequisite for the mechanical closure of pores [22,30]. This deformation exerts a compressive effect on microscopic defects within the matrix, providing contact conditions for atomic diffusion bonding [31,32]. The isostatic pressure applied to the outer surface of the capsule is transmitted through the thermally softened material matrix to the internal micro-defect interfaces. Pores gradually close during the local plastic flow and creep of the surrounding material, achieving capsule densification [4].

**Figure 4:**
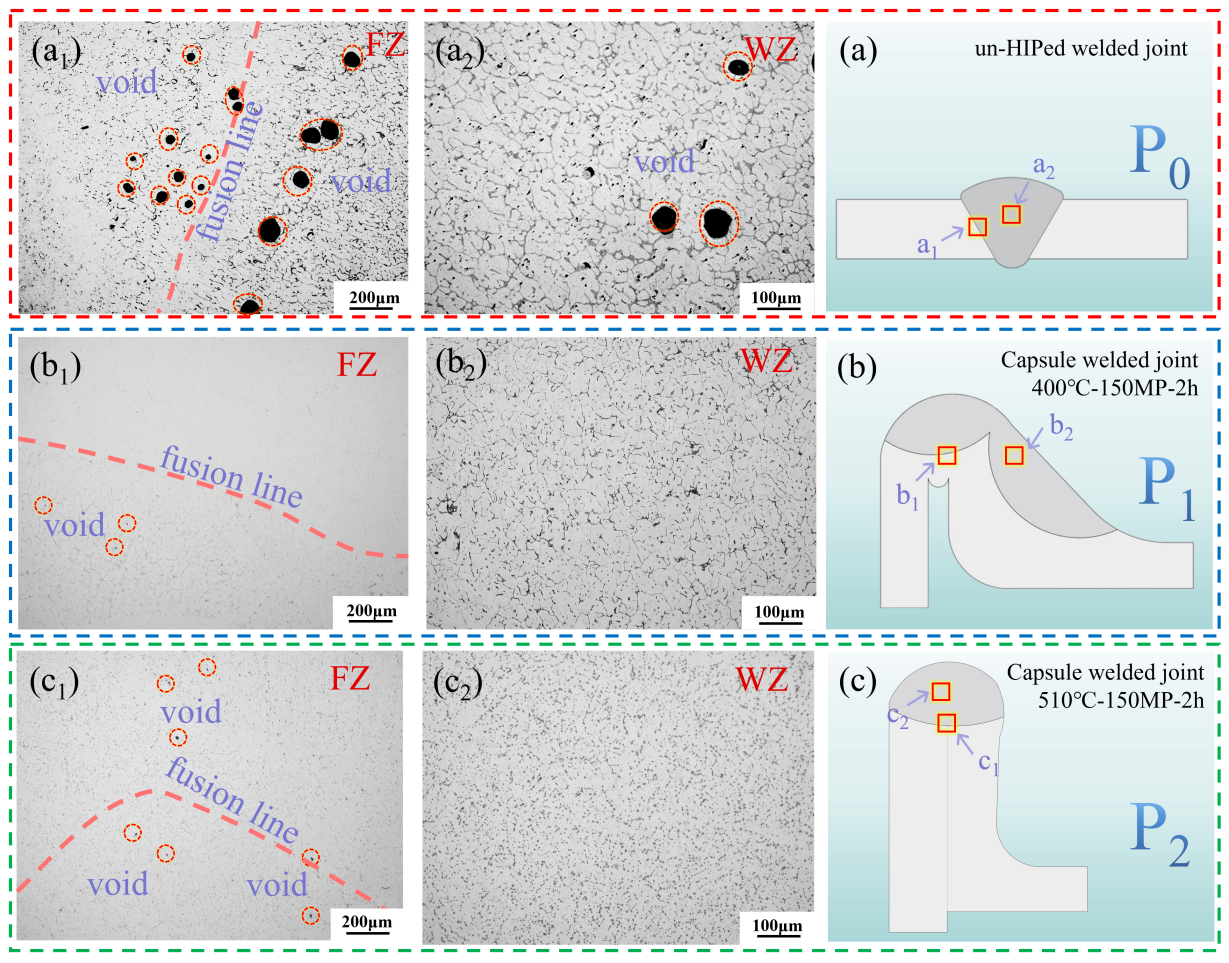
Microstructure and defect characterization of the welded joints. (**a**) Schematic of the as-welded joint without HIP; (**a_1_**) pore distribution in the P_0_ specimen; (**a_2_**) weld metal zone of the P_0_ specimen; (**b**) Schematic of the welded joint after HIP at 400 °C-150 MPa-2 h; (**b_1_**) pore distribution in the P_1_ specimen; (**b_2_**) weld metal zone of the P_1_ specimen; (**c**) Schematic of the welded joint after HIP at 510 °C-150 MPa-2 h; (**c_1_**) pore distribution in the P_2_ specimen; (**c_2_**) weld metal zone of the P_2_ specimen.

The P_0_ specimen without HIP treatment contained numerous pores of varying sizes in its weld (Figure 4a_1_,a_2_). In contrast, the P_1_ and P_2_ specimens showed a significant reduction in pore quantity and size after HIP processing (Figure 4b_1_,c_1_). This change confirms the promoting effect of macroscopic plastic deformation on pore healing. HIP densification is essentially a continuous process comprising “mechanical closure” and “diffusion bonding” [33]: in the initial stage of heating and pressurization, the capsule undergoes plastic flow and creep, and pores are mechanically compressed under isostatic pressure. During the subsequent holding stage, residual micro-pores achieve atomic-level metallurgical bonding via surface and grain boundary diffusion mechanisms. The elimination of pores not only increases the local density of the welded joint but, more importantly, removes potential stress concentration sites and preferred crack propagation paths within the joint. This fundamentally reduces the probability of hot crack formation, ensuring the overall service reliability of the capsule.

The above analysis indicates that HIP effectively eliminates pore defects in the welded joint through macroscopic plastic deformation and diffusion mechanisms, laying the foundation for improving capsule service reliability. However, the impact of the HIP process on the welded joint extends far beyond defect densification. Under the sustained action of high temperature and pressure, significant microstructural evolution occurs, primarily in two aspects: first, the morphology, type, and distribution of second phases; second, the crystallographic texture and micro-strain distribution [34,35].

The former directly determines the local strengthening mechanism and stress concentration level of the joint, while the latter affects deformation compatibility and internal stress release efficiency. Both synergistically determine the hot crack resistance and overall reliability of the capsule during service within the HIP thermo-mechanical field. Therefore, based on elucidating the pore healing behavior, it is necessary to further investigate the influence mechanism of HIP temperature on the evolution of second phases and texture, in order to fully understand the intrinsic mechanism by which the HIP process optimizes the comprehensive performance of the welded joint.

### 3.2. Evolution of Second Phases

**Figure 5:**
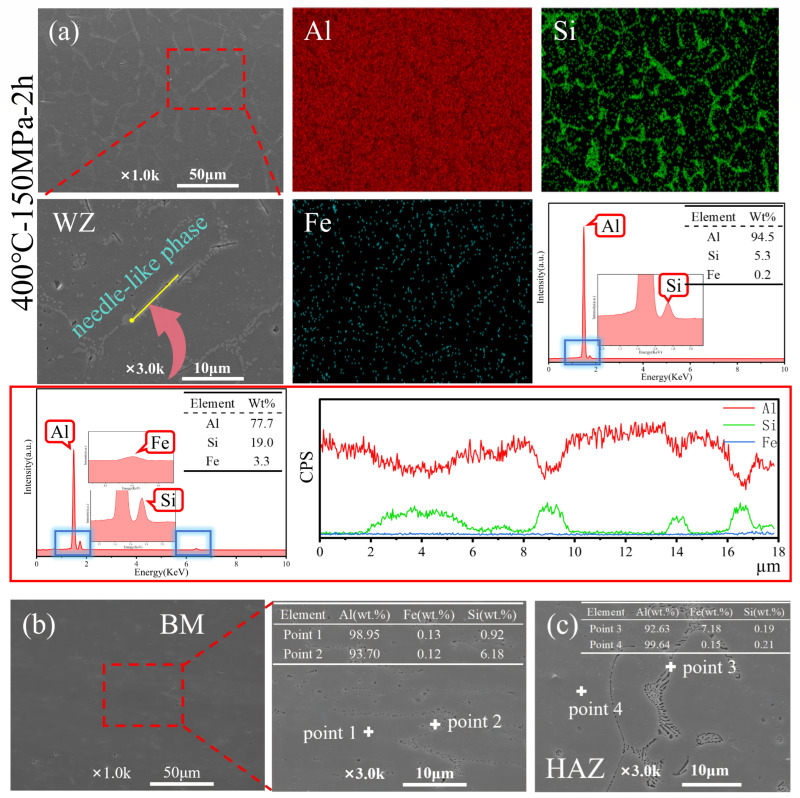
SEM image and EDS mapping of the P_1_ specimen (400 °C HIP). (**a**) Weld zone revealing the continuous Al-Si eutectic network and needle-like phases; (**b**) Base metal showing fine Fe-rich clusters; (**c**) Heat-affected zone showing Fe-rich segregation at grain boundaries.

In the P_1_ specimen treated by HIP at 400 °C, the WZ microstructure largely retained the non-equilibrium characteristics formed during weld solidification. As shown in Figure 5a, Si elements remained primarily in the original segregated regions, and the continuously distributed low-melting-point Al-Si eutectic network at grain boundaries did not undergo significant fragmentation or spheroidization. EDS mapping results indicated that the average contents of Si and Fe elements in this region were approximately 5.3 wt.% and 0.2 wt.%, respectively. High-magnification SEM observation revealed a large number of black, needle-like precipitates near grain boundaries. Line scan analysis showed Si and Fe contents of 19.0 wt.% and 3.0 wt.%, respectively.

The continuously distributed brittle Al-Si eutectic network and needle-like second phases acted as strengthening phases, effectively hindering dislocation motion and generating a significant second-phase strengthening effect. However, this strengthening mechanism, dominated by a continuous brittle network, while improving strength, also introduced significant microstructural risks. On one hand, this network structure is prone to local liquation during the HIP thermal cycle, forming liquid films at grain boundaries and weakening grain boundary cohesion [36]; On the other hand, the tips of the needle-like second phases can easily induce stress concentration at the eutectic network, and this brittle network struggles to accommodate internal stresses through plastic deformation, leading to difficulties in local stress release [19]. This coupled effect of “strengthening-stress concentration-difficulty in stress release” makes the eutectic network and needle-like phases a preferred path for crack initiation and propagation, creating a potential crack initiation hazard [19,37].

In the base metal (BM), due to the low processing temperature of 400 °C, the diffusion ability of Fe atoms was limited. As shown in Figure 5b, EDS point analysis (Point 1) of the matrix region shows Si and Fe contents of approximately 0.13 wt.% and 0.92 wt.%, respectively. Meanwhile, SEM observation reveals a small number of finely dispersed particles. Point analysis (Point 2) conducted on these particles shows a high Fe concentration (6.18 wt.%) coupled with a low Si content, more likely representing Fe-rich metastable clusters formed by supersaturated Fe atoms rather than stable AlFe-based intermetallic compounds, indicating that the BM region remained in a non-equilibrium state. The lack of effective precipitation strengthening phases resulted in low BM hardness. In the HAZ, notable compositional heterogeneity was also observed (Figure 5c), with Fe-rich segregation zones (Point 3 shows an Fe content of approximately 7.25 wt.%) present at grain boundaries. This compositional heterogeneity and interfacial instability weakened grain boundary stability, and combined with grain coarsening, made the HAZ another weak zone in the entire joint [18,38].

**Figure 6:**
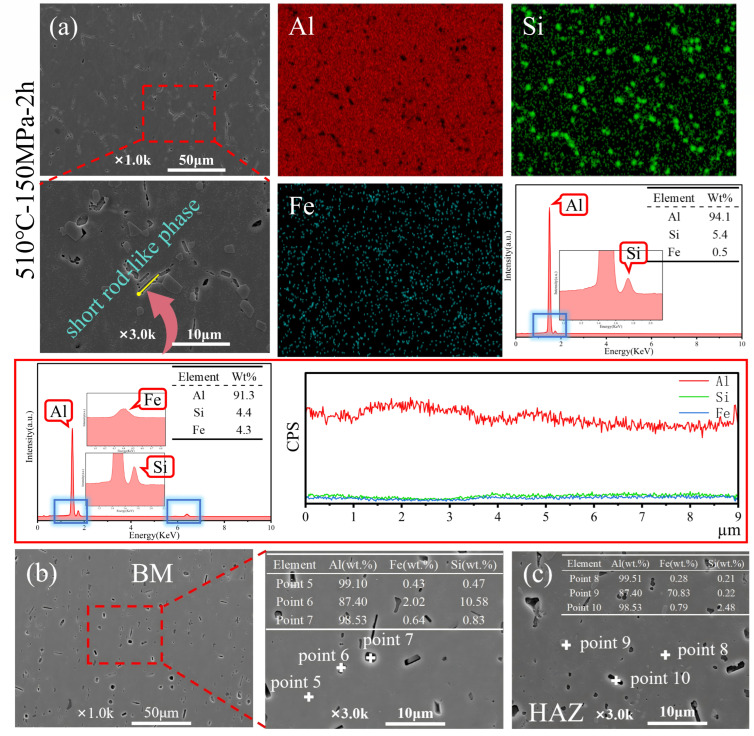
SEM image and EDS mapping of the P_2_ specimen (510 °C HIP). (**a**) Weld zone showing the spheroidized Al-Si eutectic and globular/short-rod second phases; (**b**) Base metal showing uniformly distributed globular precipitates within grains; (**c**) Heat-affected zone showing homogenized element distribution without significant Fe segregation.

When the HIP temperature was increased to 510 °C (P_2_ specimen), the welded joint microstructure underwent significant reconstruction, directly leading to a reversal of the hardness distribution. In the WZ (Figure 6a), the continuous Al-Si eutectic network transformed into granular structures dispersed along grain boundaries, with Si content of approximately 5.4 wt.% and Fe content reduced to 0.5 wt.%. Simultaneously, the original needle-like second phases transformed into thermodynamically more stable globular or short-rod precipitates, EDS line scan results show that these precipitates contain approximately 4.4 wt.% Si and 4.3 wt.% Fe.

**Figure 7:**
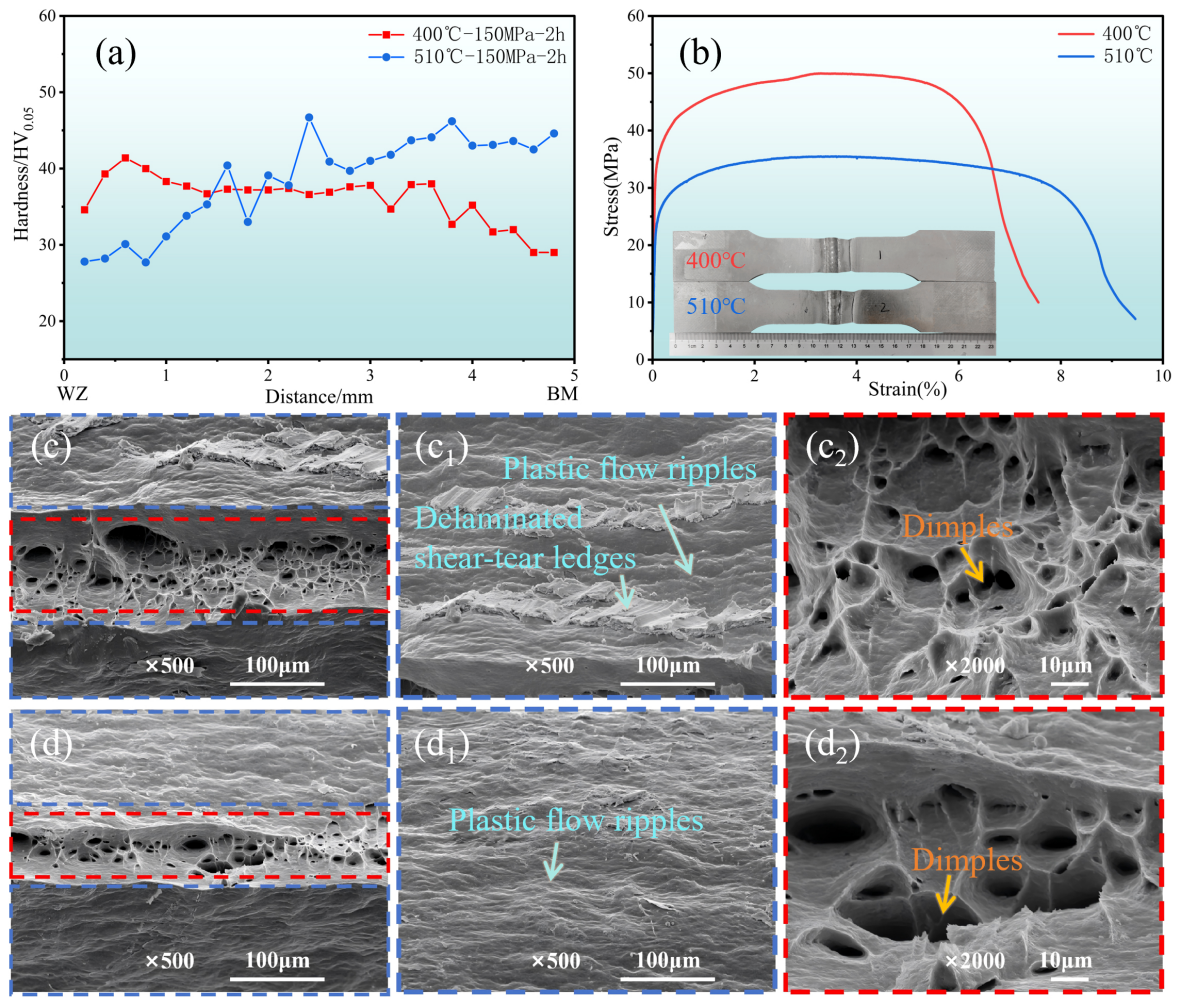
(**a**) Microhardness profiles across the welded joints under different HIP conditions; (**b**) High-temperature tensile stress–strain curves of the P_W1_ and P_W2_ specimens; (**c**,**d**) Fracture surface SEM morphologies of the P_W1_ and P_W2_ specimens; (**c_1_**,**d_1_**) Low-magnification delaminated shear-tear ledges on the fracture surface; (**c_2_**,**d_2_**) High-magnification dimple morphology.

Si atoms achieved the fragmentation and spheroidization of the eutectic network via long-range diffusion under the Ostwald ripening mechanism, transforming it into dispersed “bead-like” Si particles [27,39,40]. This morphological change significantly weakened the second-phase strengthening effect, leading to a notable decrease in WZ hardness (Figure 7a). However, this softening of the welded joint is beneficial: the bead-like structure resulting from the spheroidization of the low-melting-point Al-Si eutectic network disrupted the continuity of the brittle network, eliminating the conditions for forming continuous liquid films at grain boundaries. Furthermore, the spheroidized second phases have a blunted morphology, reducing their cutting effect on the Al matrix and effectively lowering the risk of stress concentration. The synergistic effect of these changes fundamentally reduces the risk of capsule failure.

In the BM (Figure 6b), Point 6 shows a relatively high Fe content of 10.58 wt.% and a low Si content of 2.02 wt.%. A large number of globular or short-rod second phases precipitated within the grains, characterized by high Fe content (10.58 wt.%) and low Si content (2.02 wt.%). This process indicates that, driven by high-temperature diffusion, supersaturated Fe atoms transform into stable precipitates through a dissolution-reprecipitation mechanism, thereby achieving effective precipitation strengthening. This is the fundamental reason for the significant increase in BM hardness under the 510 °C HIP treatment, surpassing that of the WZ (Figure 7a) [41]. Concurrently, the compositional heterogeneity in the HAZ was also significantly optimized (Figure 6c), with Fe segregation notably weakened and elemental distribution tending towards uniformity. This is mainly attributed to enhanced diffusion ability at high temperatures and the elimination of originally enriched interfaces by static recrystallization and grain boundary migration processes [42,43].

As discussed above, the HIP temperature alters the micro-mechanical state of the welded joint by regulating the morphology, type, and distribution of second phases. To verify the influence of temperature on macroscopic mechanical behavior, tensile tests simulating the HIP high-temperature environment were conducted. It should be clarified that this experiment only simulated the high-temperature conditions, without the application of isostatic pressure. The pressure primarily promotes macroscopic densification and pore healing (Section 3.1), while the temperature serves as the core driving force for the evolution of second phases and the recovery and recrystallization of the matrix [23]. Therefore, the high-temperature tensile results can provide important references for understanding the relationship between temperature-driven microstructural evolution and macroscopic mechanical properties.

The high-temperature tensile mechanical responses of the P_W1_ (400 °C) and P_W2_ (510 °C) specimens are shown in Figure 7b. The yield strength and ultimate tensile strength of the P_W1_ specimen were 37.22 MPa and 49.99 MPa, respectively, with an elongation after fracture of 7.6%. The P_W2_ specimen showed significantly lower strength (yield strength 28.12 MPa, ultimate tensile strength 35.50 MPa) but improved elongation after fracture (9.5%), accompanied by noticeable necking before fracture.

The fracture surface micro-morphologies are shown in Figure 7c,d. The differences in macroscopic mechanical behavior are highly coupled with the aforementioned second-phase evolution characteristics: the continuous brittle Al-Si eutectic network and needle-like second phases retained in the P_W1_ structure effectively hinder dislocation motion in the initial deformation stage, contributing to higher strength. However, such a continuous brittle network severely compromises the deformation compatibility of the structure, causing micro-void nucleation to accelerate and rapidly coalesce without undergoing sufficient plastic expansion. Consequently, the fracture surface exhibits dense, fine, shallow dimples (Figure 7c_2_), and significant delamination-like shear-tear ledges are visible at low magnification (Figure 7c_1_).

In contrast, the brittle network in the P_W2_ structure has been spheroidized into “bead-like” Si particles via the Ostwald ripening mechanism, and the needle-like second phases have transformed into blunted morphologies. The elimination of sharp stress concentration sources and the significant softening of the matrix shift the deformation mechanism towards uniform plastic deformation dominated by normal stress. The plastic flow ripples become smoother (Figure 7d_1_), and micro-voids undergo sufficient plastic expansion and coalescence, evolving into a locally concentrated, high-ductility fracture morphology dominated by large, deep dimples on the fracture surface (Figure 7d_2_).

Thus, the HIP temperature, by regulating the morphology and distribution of second phases, influences the degree of softening, micro-mechanical state, and high-temperature fracture mode of the welded joint. It is noteworthy that the evolution of fracture surface morphology further reveals that the driving factors for the abrupt change in mechanical behavior are not limited to the single evolution of the second phase interface. The undulating slip bands and delamination-like tearing on the P_W1_ fracture surface strongly suggest that the matrix retained a strong crystallographic preferred orientation and microstructural anisotropy, causing strain energy to severely concentrate in specific slip systems or at grain boundaries during deformation. The significant weakening of slip characteristics in the P_W2_ fracture indicates that a profound crystallographic reconstruction has been completed within the matrix, namely the full advancement of static recrystallization, the randomization of solidification texture, and the comprehensive release of microscopic residual strain, which endows the material with a high degree of uniformity at the lattice scale.

The differences in joint mechanical properties and fracture surface morphologies under different HIP temperatures are driven by factors beyond the simple evolution of second-phase morphology. The undulating slip bands on the P_W1_ fracture surface indicate a strong crystallographic preferred orientation and microstructural anisotropy, leading to severe localization of strain energy during deformation. The markedly weakened slip characteristics in the P_W2_ sample suggest profound microstructural reconstruction within the matrix. The abrupt change in macroscopic mechanical response is essentially an external manifestation of the synergistic evolution of dislocations, grain boundaries, and texture at the lattice scale. Macroscopic data and phase interface characterization are insufficient to capture this lattice distortion essence, necessitating a deeper exploration from the micro-crystallographic perspective.

### 3.3. Evolution of Texture

To systematically elucidate the intrinsic regulatory mechanism of different HIP temperatures on the microstructural reconstruction of welded joints, electron backscatter diffraction (EBSD) was employed to characterize the as-welded (P_0_), 400 °C HIP (P_1_), and 510 °C HIP (P_2_) joints, focusing on grain morphology, texture evolution, grain boundary characteristics, and micro-strain distribution.

**Figure 8:**
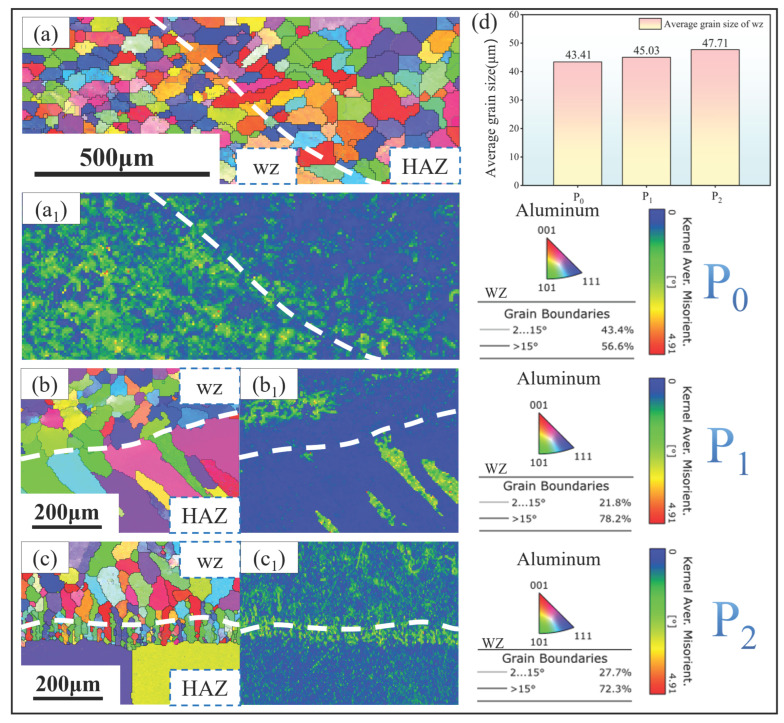
EBSD analysis of the welded joints, revealing grain morphology evolution, texture reconstruction, and strain distribution across different HIP conditions. (**a**,**a_1_**) IPF and KAM maps of the P0 specimen (as-welded, <100> texture pole density 8.72); (**b**,**b_1_**) IPF and KAM maps of the P_1_ specimen (400 °C HIP, pole density reduced to 8.25); (**c**,**c_1_**) IPF and KAM maps of the P_2_ specimen (510 °C HIP, pole density reduced to 2.50, texture eliminated); (**d**) Average grain size in the weld zone.

**Figure 9:**
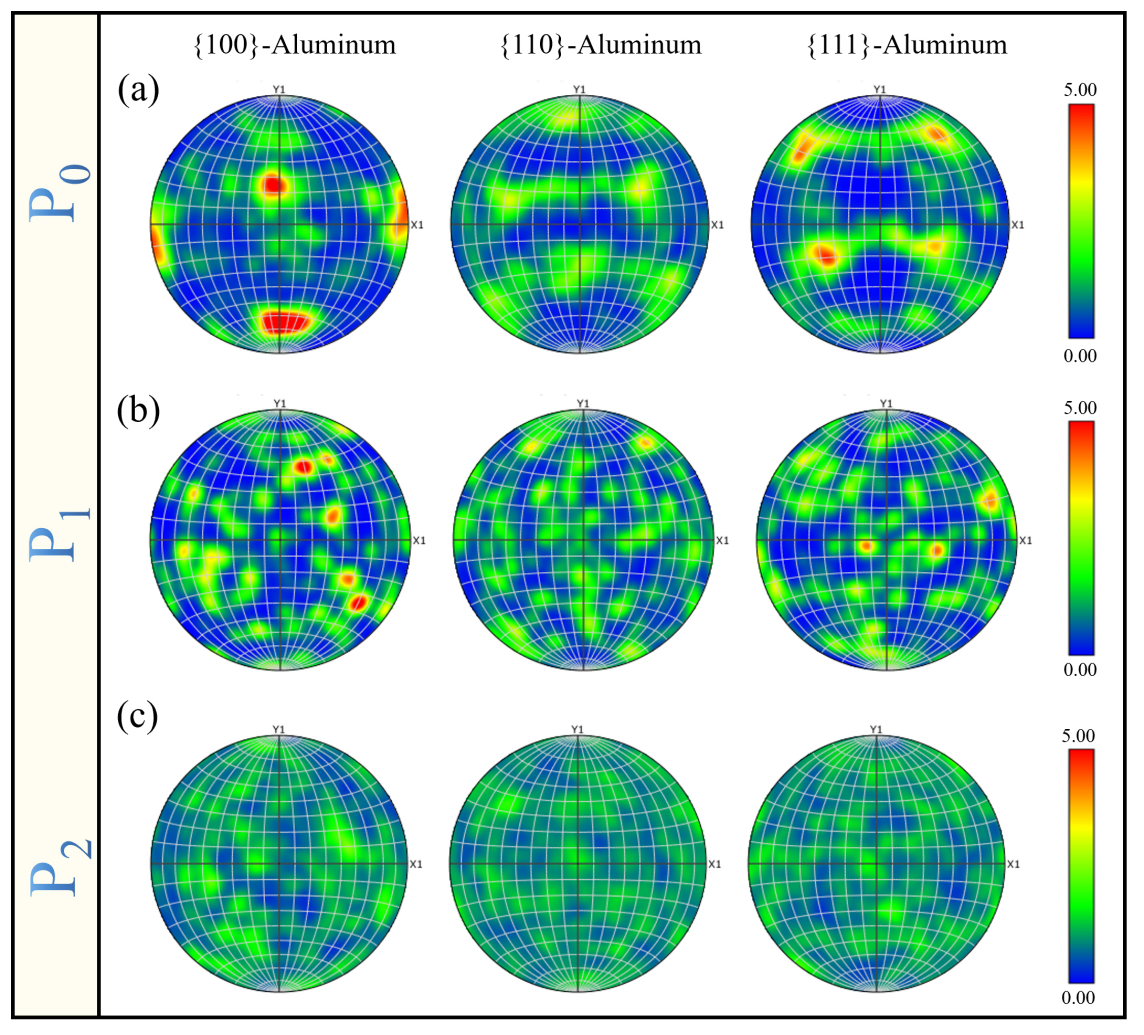
Pole figures of the welded joints processed at different HIP conditions, showing the evolution of <100> texture intensity. (**a**) P_0_ (as-welded, maximum pole density 8.72); (**b**) P_1_ (400 °C HIP, maximum pole density 8.25); (**c**) P_2_ (510 °C HIP, maximum pole density 2.50, indicating complete texture elimination).

As shown in Figure 8a, the as-welded joint (P_0_) exhibits a typical solidification microstructure. The inverse pole figure (IPF, Figure 8b) shows that the WZ is predominantly composed of equiaxed grains, but columnar grains are evident near the fusion line. These columnar grains grow perpendicular to the fusion line towards the weld center, a direct manifestation of epitaxial growth and directional solidification in the molten pool. Pole figure (PF, Figure 9a) analysis reveals a significant <100> preferred orientation texture of the Al matrix (the maximum pole density is 8.72), further confirming the preferential crystal growth along the maximum heat dissipation direction during solidification.

The Kernel Average Misorientation (KAM) map (Figure 8a_1_) shows strain energy concentrated mainly near columnar grain boundaries and the WZ/HAZ interface, primarily stemming from local stress concentrations from welding residual stress and solidification defects (e.g., porosity). Based on EBSD statistics, the proportion of low-angle grain boundaries (LAGBs, 2–15°) in the WZ was approximately 43.4%, while high-angle grain boundaries (HAGBs, >15%) accounted for about 56.6%. The microstructural feature characterized by columnar grains, a strong crystallographic texture, and high local residual strain, when combined with the continuous brittle Al-Si eutectic network, collectively constitutes the initially weak region of the joint.

After 400 °C HIP (P_1_) treatment, significant but limited changes occurred in the joint’s microstructure. The IPF map (Figure 8b) shows that the original columnar grains in the WZ were eliminated, with a modest increase in WZ grain size compared to the as-welded state (P_0_). However, the grains in the HAZ underwent noticeable coarsening, with an average grain size larger than that of the P_0_ HAZ. This evolution results from the synergistic effect of the thermo-mechanical field: thermal activation at 400 °C lowers the energy barrier for atomic diffusion and dislocation motion, providing kinetic conditions for the recovery process. Concurrently, the 150 MPa isostatic pressure provides additional strain energy as a driving force for microstructural evolution by inducing microscopic plastic deformation (creep) in the matrix and promoting pore closure.

However, in-depth texture and grain boundary analysis reveals the limitations of the microstructural evolution. The pole figure (Figure 9b) shows that although the <100> texture intensity is weakened compared to the P_0_ state (maximum pole density reduced to 8.25), the preferred orientation still clearly exists. Simultaneously, EBSD data statistics show that in the WZ of the P_1_ state, the LAGBs proportion was approximately 21.8%, and the HAGBs proportion was approximately 78.2%.

Combined with the observations from the KAM map (Figure 8b_1_), the localized high-strain regions transitioned from a dispersed distribution in the P_0_ state to localized accumulation along original grain boundaries or specific deformation bands. These pieces of evidence collectively indicate that at 400 °C, the thermo-mechanical driving force provided by the system is insufficient to drive a large-scale recrystallization process. The dominant mechanism for microstructural evolution at this stage is “pressure-assisted static recovery and limited static recrystallization”.

Based on the plastic deformation induced by isostatic pressure, thermal activation promotes dislocation slip, climb, and rearrangement, forming clearer subgrain structures (i.e., dislocation cells composed of LAGBs) and regularizing the local strain distribution. This process may be accompanied by a small number of subgrains increasing their misorientation through rotation and merging, partially transforming into HAGBs. However, it is far from reaching the extent required for complete and thorough grain boundary reorganization and texture randomization. This corroborates the conclusion from Section 3.2 that 400 °C HIP failed to effectively alter the harmful second-phase morphology, collectively demonstrating the incompleteness of microstructural modification under this process parameter.

When the HIP temperature was increased to 510 °C (P_2_), the welded joint’s microstructure underwent a fundamental reconstruction, exhibiting highly unique metallurgical phenomena. Grain size statistics (Figure 8d) show that the average WZ grain size increased only modestly compared to the P_0_ and P_1_ states, but the microstructural evolution displayed significant spatial heterogeneity. Near the fusion line, the preferential growth characteristics of columnar grains were evident, while the weld center microstructure was dominated by uniform equiaxed grains. The most critical change was the presence of an extremely fine transition grain band along the fusion line between the coarse HAZ grains and the WZ (Figure 8c). This phenomenon indicates that the P_2_ process, under the special strain gradient at this location, triggered localized recrystallization nucleation, successfully blocking the direct influence of the coarse matrix on the weld.

The most critical change is manifested in the crystallographic texture: the pole figure (Figure 9c) shows that the pole density distribution of the Al {100}, {110}, and {111} planes became highly diffuse and uniform (maximum pole density reduced to 2.50), indicating that the strong <100> solidification texture in the original WZ was essentially eliminated and tended towards randomization. This phenomenon is a hallmark that the recrystallization process is essentially complete. The temperature of 510 °C provides sufficiently high thermal activation energy to drive a static recrystallization-dominated microstructural reconstruction.

However, due to inherent differences in initial microstructure, dislocation density, interface energy, and compositional distribution across different regions of the original welded joint, their responses to the same HIP process vary. At the fusion line, the interface region with the steepest compositional and microstructural gradient, the high density of defects and interface energy provides a strong driving force for recrystallization nucleation, leading to dense nucleation and a pronounced equiaxed trend. In the HAZ, the high thermal activation energy may promote the dissolution or coarsening of second phases that pin grain boundaries, weakening the Zener pinning force. Combined with the initially lower strain energy in this region, this provides conditions for the rapid migration of a few grain boundaries and abnormal grain growth, resulting in significant coarsening.

Driven jointly by the high temperature and the stored energy accumulated from prior pressure-induced deformation, numerous strain-free new grains nucleate and grow within the deformed matrix (e.g., regions of high dislocation density, original grain boundaries, and the fusion line interface), eventually consuming the original deformed structure and forming a new grain structure with a nearly random orientation overall.

The large areas of low strain (blue) in the KAM map (Figure 8c_1_) and the significant reduction in the average KAM value confirm that the recrystallization process effectively eliminated dislocations and released micro-strain. Grain boundary statistics provide further evidence for this transformation: the proportion of HAGBs further increased to 72.3%, while the proportion of LAGBs was further compressed. This three-part characteristic of “randomized texture, HAGB dominance, and micro-strain release” is a classic microstructural signature of a material that has undergone complete recrystallization.

The recrystallized microstructure characteristics revealed by this EBSD analysis corroborate the second-phase morphology evolution observed by SEM in Section 3.2, collectively elucidating the micro-mechanism for the performance improvement of the welded joint after 510 °C HIP. Specifically, the recrystallization process fundamentally releases micro-strain accumulated from welding and deformation [44], significantly reducing the internal stress level of the material.

Simultaneously, the full spheroidization of second phases disrupts the original continuous brittle network, eliminating sharp stress concentration sources. The macroscopic release of internal stress and the elimination of microscopic stress concentration are synergistic optimization effects produced by the same HIP process at the lattice scale and phase interface scale. Although microstructural spatial heterogeneity persists, implying a gradient in mechanical properties, this overall optimization of the microstructure fundamentally harmonizes the balance between strength, plasticity, and toughness, thereby determining its superior macroscopic mechanical behavior.

**Figure 10:**
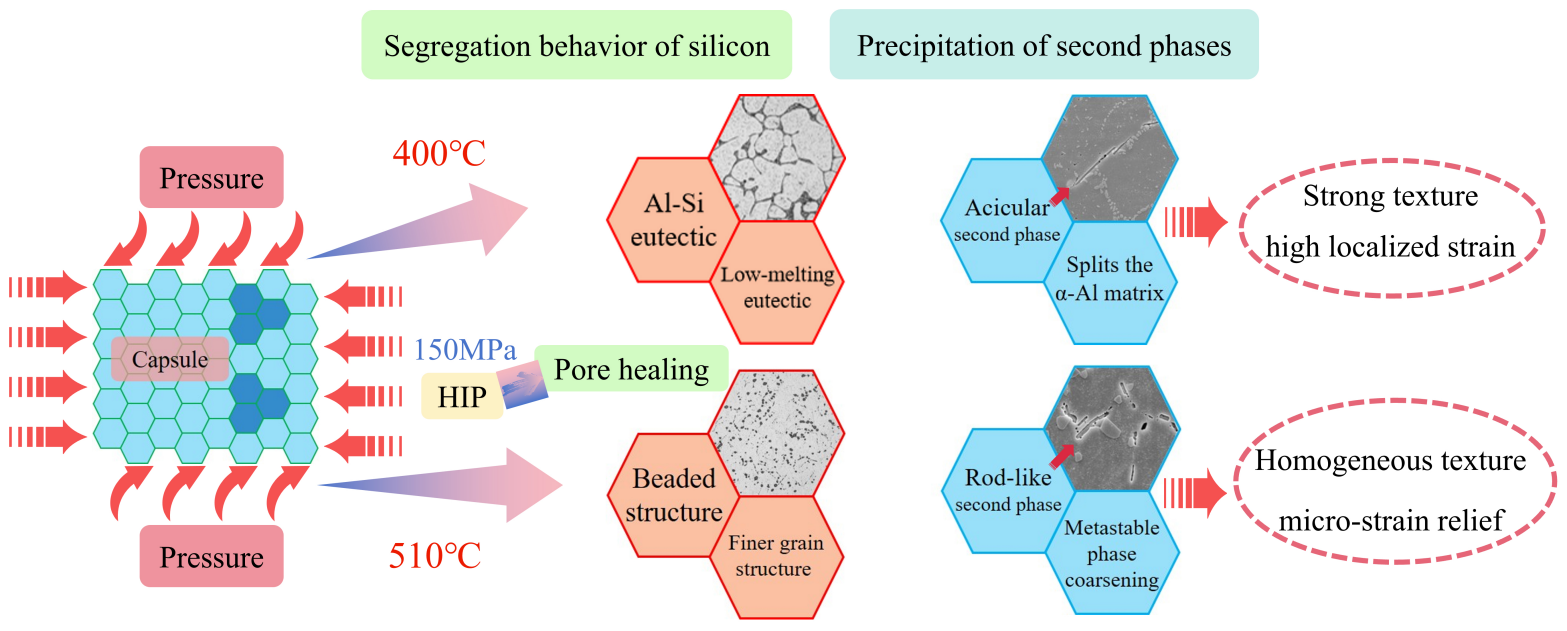
Schematic diagram summarizing the capsule, second-phase, and texture evolution during HIP: 400 °C HIP retains the continuous Al-Si eutectic network and needle-like phases with partial texture weakening (pressure-driven densification), while 510 °C HIP drives complete Al-Si eutectic spheroidization, second-phase coarsening, full recrystallization, and texture elimination (thermally driven reconstruction).

In summary, HIP temperature is the key factor governing the microstructure and properties of 1060Al welded joints. Figure 10 illustrates the relationship between second-phase and texture evolution at different HIP temperatures. The 400 °C HIP is primarily pressure-driven, achieving densification and limited recovery but retaining high strain and partial texture. The 510 °C HIP provides sufficient thermal activation, driving complete recrystallization, leading to randomized texture, increased HAGB proportion, and release of micro-strain. This synergizes with the spheroidized second phases, laying the microstructural foundation for improved overall performance.

## 4. Conclusions

This paper systematically studied the effects of two typical HIP temperatures on the second phases and texture of 1060 aluminum alloy TIG welded joints, revealing the thermo-mechanical coupling mechanism that governs the synergistic evolution of second phases and texture under different thermal activation conditions. The main conclusions are as follows:

(1) Dual effect of HIP temperature: The 400 °C HIP treatment is dominated by pressure-driven densification. Although it heals pores, the insufficient thermal activation limits microstructural evolution. Under this condition, the WZ retains a continuous low-melting-point Al-Si eutectic network and needle-like second phases, which act as stress concentration sources. Quantitatively, the <100> texture maximum pole density only decreased from 8.72 to 8.25, and local strain remained concentrated along original grain boundaries, confirming that the dominant mechanism is pressure-assisted static recovery rather than complete recrystallization.
(2) Synergistic optimization of second phases and texture: At 510 °C, the synergy of stored deformation energy and sufficient thermal activation drives a dual microstructural reconstruction. The Al-Si eutectic undergoes Ostwald ripening into a “bead-like” structure, and needle-like second phases transform into short-rod/globular morphologies, eliminating grain boundary liquid films and sharp stress raisers. Concurrently, the matrix undergoes complete static recrystallization: the maximum pole density drops to 2.50, the HAGB proportion reaches 72.3%, and the high-temperature elongation increases from 7.6% to 9.5%, indicating a fracture mode transition from localized shear tearing to uniform plastic deformation. This phase-interface and lattice-scale synergistic optimization inherently mitigates the microstructural failure driving forces of thin-walled capsules.

## Figures and Tables

**Table 1:** Chemical compositions of 1060Al and ER1070 (wt.%).

Elements (wt.%)	Al	Si	Fe	Cu	Mg	Zn	Mn	Ti	V
1060Al	99.60	0.25	0.35	0.05	0.03	0.05	0.03	0.03	0.05
ER1070	99.70	0.20	0.25	0.04	0.03	0.04	0.03	0.03	0.05

**Table 2:** Processing parameters of HIP and high-temperature tensile testing.

Sample No.	Temp. (℃)	Pressure (MPa)	Hold Time (h)	Test No.	Temp. (℃)	Strain Rate (s^−1^)
P_1_	400	150	2	P_W1_	400	3.0 × 10^−4^
P_2_	510	150	2	P_W2_	510	3.0 × 10^−4^

## Data Availability

The data presented in this study are available on request from the corresponding author, as the data are still being used for ongoing follow-up research.
